# Transportin-1: A Nuclear Import Receptor with Moonlighting Functions

**DOI:** 10.3389/fmolb.2021.638149

**Published:** 2021-02-18

**Authors:** Allegra Mboukou, Vinod Rajendra, Renata Kleinova, Carine Tisné, Michael F. Jantsch, Pierre Barraud

**Affiliations:** ^1^ Expression Génétique Microbienne, Institut de Biologie Physico-Chimique (IBPC), UMR 8261, CNRS, Université de Paris, Paris, France; ^2^ Department of Cell and Developmental Biology, Center for Anatomy and Cell Biology, Medical University of Vienna, Vienna, Austria

**Keywords:** karyopherin, karyopherin-β2, nucleo-cytoplasmic transport, NLS, proline–tyrosine nuclear localization signal, phase-separation, FUS, TNPO1

## Abstract

Transportin-1 (Trn1), also known as karyopherin-β2 (Kapβ2), is probably the best-characterized nuclear import receptor of the karyopherin-β family after Importin-β, but certain aspects of its functions in cells are still puzzling or are just recently emerging. Since the initial identification of Trn1 as the nuclear import receptor of hnRNP A1 ∼25 years ago, several molecular and structural studies have unveiled and refined our understanding of Trn1-mediated nuclear import. In particular, the understanding at a molecular level of the NLS recognition by Trn1 made a decisive step forward with the identification of a new class of NLSs called PY-NLSs, which constitute the best-characterized substrates of Trn1. Besides PY-NLSs, many Trn1 cargoes harbour NLSs that do not resemble the archetypical PY-NLS, which complicates the global understanding of cargo recognition by Trn1. Although PY-NLS recognition is well established and supported by several structures, the recognition of non-PY-NLSs by Trn1 is far less understood, but recent reports have started to shed light on the recognition of this type of NLSs. Aside from its principal and long-established activity as a nuclear import receptor, Trn1 was shown more recently to moonlight outside nuclear import. Trn1 has for instance been caught in participating in virus uncoating, ciliary transport and in modulating the phase separation properties of aggregation-prone proteins. Here, we focus on the structural and functional aspects of Trn1-mediated nuclear import, as well as on the moonlighting activities of Trn1.

## 1 Introduction

In eukaryotic cells, the presence of a physical separation between the nucleus and the cytoplasm in the form of a double membrane assures a physical and temporal separation of the transcription and translation processes but creates the need for a selective and efficient transport of thousands of macromolecules across the nuclear envelope (Görlich and Kutay, 1999; Conti and Izaurralde, 2001; Fried and Kutay, 2003; Cook et al., 2007). This continual ballet, with controlled bidirectional flows, is orchestrated by nuclear transport receptors (NTRs) that carry their cargoes from one compartment to the other by crossing the nuclear envelope at the level of the nuclear pore complexes (NPCs) (Tran and Wente, 2006; Wente and Rout, 2010; Allegretti et al., 2020). Transport receptors of the karyopherin-β (Kapβ) family account for the vast majority of the cargo flow through the NPC. Karyopherins interact selectively with proteins of the NPC, namely the phenylalanine-glycine nucleoporins (FG-Nups), which surround and line the NPC central channel (Hampoelz et al., 2019). Within the NPC, these FG-Nups built and establish the proper operation of the permeability barrier (Fu et al., 2018). In general, large macromolecules (>40 kDa) are indeed excluded from the NPC channel, whereas karyopherins can cross the NPC barrier on account of their common property to selectively interact with FG-Nups (Rout et al., 2003; Weis, 2007; Hülsmann et al., 2012; Beck and Hurt, 2017).

Karyopherins that mediate nuclear import are also known as importins, whereas those mediating nuclear export are known as exportins. Besides the binding to FG-Nups, importins and exportins associate with their cargoes via signals, namely nuclear localization signals (NLSs) and nuclear export signals (NESs), which determine whether the cargo is imported in or exported out of the nucleus (Chook and Süel, 2011; Xu et al., 2010; Cook and Conti, 2010). Importins bind to their cargoes in the cytoplasm, reach the NPCs and translocate to the other side of the nuclear envelope, where they release their cargoes upon binding to the small GTPase Ran in its GTP-bound form (RanGTP). In a reciprocal manner, exportins associated with RanGTP bind to their cargoes in the nucleus, reach the NPCs and translocate to the other side of the nuclear envelope, where they dissociate from their cargoes upon GTP hydrolysis and RanGDP release (Conti and Izaurralde, 2001; Fried and Kutay, 2003; Weis, 2003). Transport directionality is thus primarily driven by the RanGTPase nucleotide cycle, which produces an asymmetric distribution of RanGTP and RanGDP on both sides of the nuclear envelope, with RanGTP being present at high concentrations in the nucleus and with RanGDP being mainly present in the cytoplasm (Izaurralde et al., 1997; Dasso, 2002). Transport selectivity, on the other hand, relies on the selective binding of NTRs to their cargoes via the selective recognition of their signals.

Within the Kapβ family, Transportin-1 (Trn1), also known as karyopherin-β2 (Kapβ2), is probably the best-characterized nuclear import receptor after Importin-β [also known as karyopherin-β1 (Kapβ1)]. Despite nearly identical structures, Trn1 shows only 24% sequence similarity to Importin-β (Cingolani et al., 1999; Chook and Blobel, 1999). They share a higher similarity at the level of their N-terminal half where the binding of RanGTP takes place, and differ deeply at the level of their C-terminal half, the site of binding to their respective cargoes. Whereas Importin-β binds via the adaptor protein Importin-α to cargoes harbouring the ‘classical’ NLS (cNLS), Trn1 binds directly to its cargoes without adaptor (Chook and Süel, 2011; Cook et al., 2007; Twyffels et al., 2014). Trn1 has been initially identified as the nuclear import receptor of the heterogeneous nuclear ribonucleoprotein A1 (hnRNP A1) (Pollard et al., 1996; Fridell et al., 1997; Bonifaci et al., 1997). The NLS sequence of hnRNP A1 [designated the ‘M9’ sequence (Siomi and Dreyfuss, 1995; Weighardt et al., 1995)] can also mediate its nuclear export (Michael et al., 1995). Therefore, considering that the newly identified NTR could both transport hnRNP A1 in and out of the nucleus, this karyopherin was named ‘transportin’ in opposition to the names ‘importins’ and ‘exportins’ in use for unidirectional transport receptors. Trn1 was in fact later shown to only function in nuclear import, with the hnRNP A1 cargo being released from Trn1 upon RanGTP binding (Siomi et al., 1997; Izaurralde et al., 1997), a common trait of nuclear import receptors, but the terminology has remained.

In the Kapβ family, Trn1 shares the highest sequence identity (83%) with Transportin-2 (Trn2), also known as karyopherin-β2B (Kapβ2B) (Twyffels et al., 2014). Trn2 exists in two isoforms called Trn2A or Kapβ2B(A) (Siomi et al., 1997) and Trn2B or Kapβ2B(B) (Shamsher et al., 2002), the expression of which results from an alternative splicing event (Rebane et al., 2004). This high sequence similarity between Trn1 and Trn2 correlates with a highly similar cargo spectrum for these two NTRs. Among different karyopherins, Trn1 indeed presents the highest degree of functional redundancy with Trn2 in terms of cargoe specificity (Mackmull et al., 2017; Kimura et al., 2017). Their cargoes include many RNA-binding proteins involved in mRNA processing, such as proteins of the hnRNP family (e.g., hnRNP A1, hnRNP A2/B1, hnRNP D, hnRNP F, hnRNP H, hnRNP M), but also other protein families implicated in nucleic-acid-related functions in the nucleus. Although the exclusive import of specific cargoes by either Trn1 or Trn2 have not been experimentally confirmed, a high-throughput study suggested a slight specialization of these NTRs. Cargoes with efficient NLSs would be imported by any of the two Transportins, whereas cargoes with less efficient NLSs might be exclusively carried by either Trn1 or Trn2 (Kimura et al., 2017). For instance, actin and actin-related proteins implicated in chromatin remodeling and transcription, and proteins related to nuclear division, would preferentially be imported by Trn1, whereas proteins related to DNA repair would preferentially be Trn2 cargoes (Kimura et al., 2017). These recent studies aiming at defining the cargo spectrum of individual NTRs clearly provide insightful information on NTR-cargo recognition. However, these studies must be analyzed with caution, since among the few hundreds of potential Trn1 cargoes identified in two recent reports, only ∼20 are common to the two datasets (Mackmull et al., 2017; Kimura et al., 2017; Baade and Kehlenbach, 2019). The need for experimental validation would therefore be essential to understand in detail the cargo-specificity of Trn1 and to establish the potential specialization of Trn1 and Trn2.

Aside from its principal and long-established activity as a nuclear import receptor, Trn1 was shown more recently to moonlight outside nuclear import. Trn1 has been for instance caught in participating in virus uncoating, cellular cilia formation and in modulating the phase separation properties of aggregation-prone proteins. In this review, we will focus on the structural and functional aspects of Trn1-mediated nuclear import, as well as on the moonlighting activities of Trn1.

## 2 Transportin-1: A Nuclear Import Receptor

### 2.1 Structural Organization of Trn1

**FIGURE 1:**
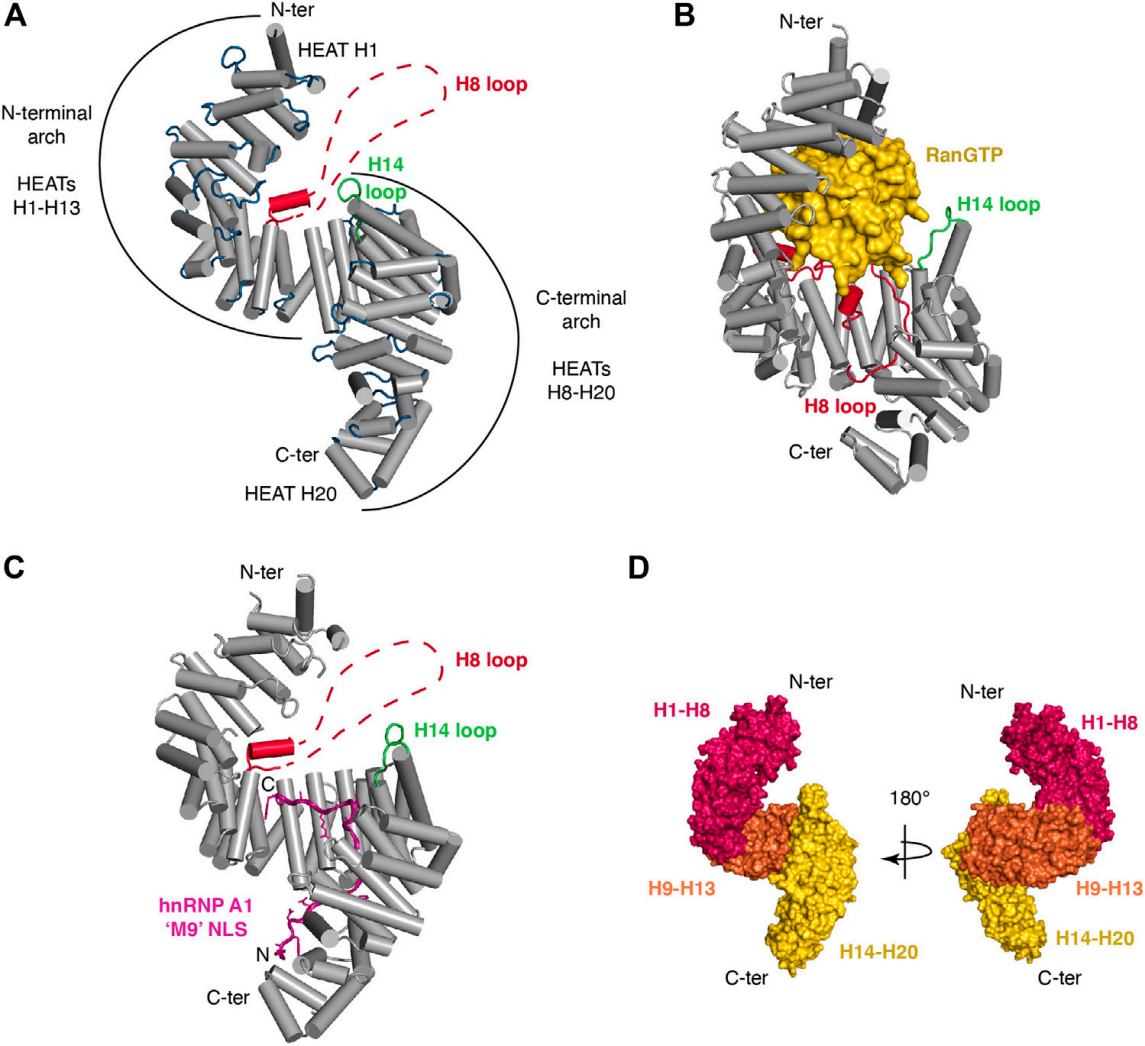
Structural organization of Transportin-1. **(A)** Trn1 is composed of 20 HEAT repeats and adopts a superhelical architecture [free Trn1: PDB code 2QMR (Cansizoglu and Chook, 2007)]. Helices of the HEAT repeats are displayed as cylinders in gray. The loops connecting the different helices are in blue, except for the loop of HEAT H14 that is in green and for the long acidic loop of HEAT H8 (H8 loop) that is in red. Most of the H8 loop is unstructured in the free Trn1 structure, and this long loop is schematically represented with a dashed line. Trn1 structure is overall formed by two consecutive and overlapping arches. The N-terminal arch consists of HEATs H1-H13, and the C-terminal arch is formed by HEATs H8-H20. **(B)** RanGTP (in yellow) associates with Trn1 (in gray) and fits snugly into the N-terminal arch [Trn1-RanGTP complex: PDB code 1QBK (Chook and Blobel, 1999)]. Upon binding, the H8 loop (in red) is reorganized and becomes almost entirely structured. **(C)** The hnRNP A1 ‘M9’ NLS (in purple) associates with Trn1 (in gray) in an extended conformation that covers almost entirely the C-terminal arch [Trn1-NLS complex: PDB code 2H4M (Lee et al., 2006)]. As in free Trn1, most of the H8 loop is unstructured in the NLS-bound structure of Trn1. **(D)** The three major segments identified to describe the conformational flexibility of Trn1 are depicted with three distinct colors, namely HEATs H1-H8 in red, H9-H13 in orange, and H14-H20 in yellow. These segments are displayed on Trn1 surface using two 180°-views to help better grasp the superhelical character of this HEAT-repeat protein.

Like other Kapβ, Trn1 is composed of HEAT-repeats and adopts an overall superhelical architecture (Figure 1). HEAT-repeats consist of tandem repeats of a ∼40-residue motif (Andrade et al., 2001) that was first identified in Huntingtin, Elongation factor 3, the regulatory A subunit of protein phosphatase 2A and the TOR lipid kinase (Andrade and Bork, 1995). Each HEAT motif folds into a pair of α-helices (known as the A and B helices). Consecutive HEAT motifs pile together in a nearly parallel fashion, which lead to an overall superhelical architecture. The inner concave surface of Trn1 is formed by the B helices, whereas the A helices are exposed at the outer convex surface. The different structures of Trn1, either free or in complex with its different partners, namely RanGTP or various NLSs (see Table 1), all showed that Trn1 is composed of 20 HEAT repeats.

**TABLE 1:** Crystal structures of Transportin-1.

Transportin-1	Partner	Type of NLS	PDB code	References
Trn1-FL	RanGTP		1QBK	(Chook and Blobel, 1999)
Trn1-ΔH8	hnRNP A1 NLS (‘M9’ NLS)	hPY-NLS	2H4M	(Lee et al., 2006)
Trn1-ΔH8	hnRNP M NLS	bPY-NLS	2OT8	(Cansizoglu et al., 2007)
Trn1-FL	–		2QMR	(Cansizoglu and Chook, 2007)
Trn1-FL	–		2Z5J	(Imasaki et al., 2007)
Trn1-FL	TAP/NXF1 NLS	PY-NLS	2Z5K, 2Z5M	(Imasaki et al., 2007)
Trn1-FL	hnRNP D NLS	bPY-NLS	2Z5N	(Imasaki et al., 2007)
Trn1-FL	hnRNP DL/JKTBP NLS	PY-NLS	2Z5O	(Imasaki et al., 2007)
Trn1-ΔH8	FUS NLS	hPY-NLS	4FDD	(Zhang and Chook, 2012)
Trn1-FL	FUS NLS	hPY-NLS	4FQ3	(Niu et al., 2012)
Trn1-ΔH8	ScNab2 NLS	∼PY-NLS	4JLQ	(Soniat et al., 2013)
Trn1-ΔH8	HCC1 NLS	PY-NLS	4OO6	–
Trn1-ΔH8	histone H3 NLS	non-PY-NLS	5J3V	(Soniat and Chook, 2016)
Trn1-ΔH8	RPL4	PY-NLS	5TQC	(Huber and Hoelz, 2017)
Trn1-ΔH8	FUS NLS	hPY-NLS	5YVG, 5YVH, 5YVI	(Yoshizawa et al., 2018)

Trn1-FL: construct of Trn1 full-length. Trn1-ΔH8: construct of Trn1 with a shortened H8 loop. hPY-NLS: hydrophobic PY-NLS. bPY-NLS: basic PY-NLS. PY-NLS: PY-NLS without a basic or hydrophobic stretch seen to interact with Trn1 in the structure. ∼PY-NLS: variation to the PY-NLS motif.

As in other Kapβ proteins, the HEAT motifs in Trn1 are connected to each other via small loops or small helices. In addition, within each HEAT motif, helix A is connected to helix B via a small loop of ∼2–4 residues, except at the level of HEAT H14, where this loop is slightly longer and consists of 9 residues, and notably also at the level of HEAT H8, where the two helices of the motif are connected via a long loop of about sixty residues (Figure 1A). This long loop, thereafter called the H8 loop, is intrinsically disordered and has an overall negative charge (27 Asp/Glu over 65 residues). A longer H8 loop is also present in other Kapβ, such as Importin-β, but it is significantly longer in Trn1 (65 vs. 12 residues in Importin-β) (Cingolani et al., 1999; Chook and Blobel, 1999; Cook et al., 2007).

Structural and functional considerations have led to define Trn1 as formed by two consecutive and overlapping arches. The N-terminal arch consists of HEATs H1-H13, and the C-terminal arch is formed by HEATs H8-H20 (Figure 1A). The N-terminal arch is the site of interaction with RanGTP (Chook and Blobel, 1999), whereas the C-terminal arch is the site of NLS recognition, as seen for the ‘M9’ sequence of hnRNP A1 (Lee et al., 2006) (Figures 1B,C).

Conformational flexibility is a hallmark of the Kapβ family of proteins (Conti et al., 2006). In Trn1, this intrinsic structural flexibility, which presumably allows Trn1 to adapt its conformation for binding its different partners, namely FG-Nups, RanGTP and various NLSs, has been initially investigated at low-resolution using small-angle X-ray scattering (SAXS) (Fukuhara et al., 2004). Later, with a growing number of Trn1 structures (Table 1), either free (Cansizoglu and Chook, 2007), or in complex with RanGTP (Chook and Blobel, 1999) and the ‘M9’ NLS of hnRNP A1 (Lee et al., 2006), the conformational flexibility of Trn1 has been analyzed at atomic-resolution using structure coordinates and some atomic-refinement parameters (namely the crystallographic B-factors) of free Trn1 (Cansizoglu and Chook, 2007). In addition, the conformational flexibility of Trn1 has been analyzed *in silico* using molecular dynamics simulations (Wang et al., 2012). Together, these studies have shown that the behaviour in terms of conformational flexibility is not uniform along the Trn1 structure, and that substructures or segments can be distinguished for describing the overall conformational flexibility of Trn1. The conformational flexibility of Trn1 has therefore been reported as ‘segmental’, with three major segments composed of HEATs H1-H8, H9-H13, and H14-H20 (Figure 1D). The two latest segments are relatively rigid substructures that rotate around a flexible hinge at the level of H13-H14 helices. The first segment is intrinsically more heterogeneous and can be subdivided into two smaller segments, the segment H1-H4, which is highly prone to conformation changes, and the segment H5-H8, the conformation of which is mostly sensitive to RanGTP binding (Cansizoglu and Chook, 2007; Wang et al., 2012). Within the segment H14-H20, repeats H19-H20 are the most prone to conformational changes.

The conformational flexibility of Trn1 is conferred by the repetition of the HEAT motifs, the packing of which produces a spring-like effect. In the different structures (Table 1), Trn1 is more stretched or more compact, and this occurs mostly at the level of its N- and C-terminal flexible repeats (H1-H4 and H19-H20). This gives an overall structure that can be more or less extended. In the structures and the dynamic simulation, free Trn1 is the one adopting the most elongated conformation, whereas the RanGTP- and the ‘M9’-NLS-bound forms are more compact (Cansizoglu and Chook, 2007; Wang et al., 2012). It is worth noting that longer loops connecting helices A and B are found at the level of the three main segment bounds, namely the H8 loop and the H14 loop. These two loops and the surrounding regions constitute dynamic ‘hotspots’ important for Trn1 function (Wang et al., 2012), namely cargo binding and its release upon binding to RanGTP (see below).

### 2.2 Trn1-Mediated Import Pathway

**FIGURE 2:**
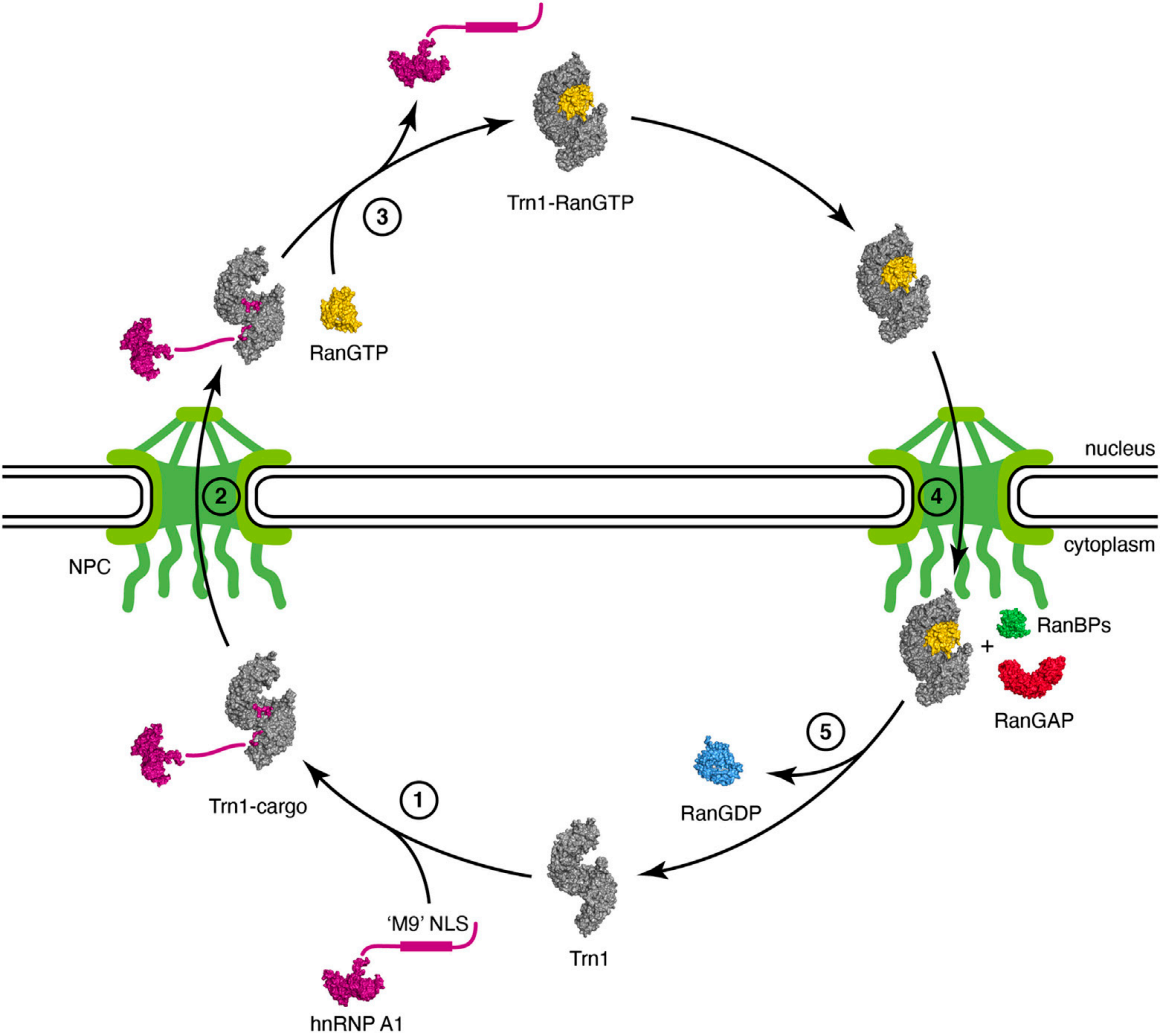
Nuclear import cycle of Transportin-1. (step 1) Trn1 associates with its cargo via the NLS in the cytoplasm [free Trn1 in gray: PDB code 2QMR (Cansizoglu and Chook, 2007); hnRNP A1 cargo with its unstructured ‘M9’ NLS in purple: PDB code 2LYV (Barraud and Allain, 2013); Trn1-cargo in gray and purple: PDB code 2H4M (Lee et al., 2006)]. (step 2) Trn1 associated with its cargo transits towards and passes through the NPC. The NPC, with the nuclear basket and the cytoplasmic filaments, is schematically represented in green. (step 3) Trn1 associated with its cargo binds to RanGTP in the nucleus, which triggers cargo release [RanGTP in yellow: PDB code 1RRP (Vetter et al., 1999); Trn1-RanGTP in gray and yellow: PDB code 1QBK (Chook and Blobel, 1999)]. (step 4) Trn1 associated with RanGTP transits towards and passes through the NPC. (step 5) At the cytoplasmic face of the NPC, RanGTP is hydrolyzed and released from Trn1 upon the combined action of RanBPs and RanGAP (RanBP1 in green and RanGAP in red: PDB code 1K5D (Seewald et al., 2002); RanGDP in blue: PDB code 1BYU (Stewart et al., 1998)]. Trn1 is free again in the cytoplasm and ready for another import cycle.

The first step of the Trn1 import cycle consists in the recognition and binding of the NLS of a Trn1 cargo in the cytoplasm (Figure 2, step 1). NLS recognition by Trn1 will be described in detail in the following subsections, but overall, binding occurs in the C-terminal arch of Trn1, as seen for the ‘M9’ sequence of hnRNP A1 (Lee et al., 2006) (Figure 1C). In the cytoplasm, RanGTP is scarce and binding of Trn1 to its cargoes occurs in its free form.

Then, Trn1 associated with its cargo via the NLS transits towards the NPCs, where its biochemical properties and ability to interact with FG-Nups allows it to pass through the NPC selectivity barrier (Figure 2, step 2).

In the nucleus, RanGTP is abundant, and cargo-associated Trn1 binds to RanGTP, which triggers cargo release (Figure 2, step 3). RanGTP binds Trn1 at the concave surface of the N-terminal arch, and more precisely at the level of the segments H1-H4 and H7-H8 but also at the level of the long and acidic H8 loop (Chook and Blobel, 1999) (Figure 1B). Parts of the ‘switch I’ and ‘switch II’ regions of Ran (switch I: residues 30–47; switch II: residues 65–80), which experience large conformational changes during the interconversion between the different nucleotide states of Ran (Vetter et al., 1999; Scheffzek et al., 1995; Cook et al., 2007), are recognized at the level of the H1-H3 segment of Trn1. Similarly to what is observed in Impβ, the basic patch of Ran (residues 139–142), located opposite to the switch regions of Ran, is involved in electrostatic interactions with HEAT repeat H7 in Trn1, but not directly with the acidic H8 loop (Lee et al., 2005). Although the RanGTP-bound structure of Trn1 is less elongated than the free Trn1 structure, the associated structural change is only minor. In contrast, binding of RanGTP to Trn1 has pronounced and extensive effects on the conformation of the H8 loop (Figures 1A,B). The long H8 loop, which is almost entirely disordered in the free Trn1 structure, becomes indeed well-structured upon interacting with RanGTP. About one third of the H8 loop residues make direct contacts with RanGTP. These contacts include both hydrophobic interactions with apolar side-chains and aromatics, and electrostatic interactions with polar and charged side-chains (Chook and Blobel, 1999). The part of the H8 loop that does not make direct contact with RanGTP adopts an extended conformation and interacts extensively with the segment H12-H18 in the C-terminal arch (Figure 1B). The H8 loop thereby occupies the NLS interaction sites as observed for the ‘M9’ sequence of hnRNP A1 (Figure 1C). The interaction of the loop with the NLS-binding site depends on RanGTP, and the displacement and structuring of the H8 loop at the NLS-binding site provides a molecular mechanism to the cargo-dissociation upon RanGTP binding (Lee et al., 2006; Chook and Blobel, 1999). The involvement of the H8 loop in cargo dissociation is also supported by the fact that its removal allows Trn1 to bind the ‘M9’ NLS and RanGTP simultaneously (Chook et al., 2002). By investigating the binding and dissociation of several NLSs, the mechanism of substrate release from Trn1 has been refined and was reported to occur in a stepwise manner. Upon RanGTP binding, NLS dissociation would first occur at the level of the segment H14-H18, also called ‘site B’, and complete release of the cargo would then occur upon dissociation of the NLS from the segment H8-H13, also called ‘site A’ (Imasaki et al., 2007).

Then, Trn1 associated with RanGTP transits towards the NPCs, and interacts with FG-Nups to pass through the NPC selectivity barrier, similarly to its entry into the nucleus, but in the opposite direction (Figure 2, step 4).

When Trn1 bound to RanGTP reaches the cytoplasmic face of the NPC, it associates with several cytoplasmic factors involved in RanGTP hydrolysis and dissociation from the karyopherin, namely the Ran GTPase-activating protein (RanGAP) and the Ran-binding proteins RanBP1 and RanBP2. RanBP2, also known as nucleoporin 358 (Nup358), harbors four Ran-binding domains (RanBD) and is a component of the cytoplasmic filaments of the NPC (Delphin et al., 1997). The intrinsic GTP-hydrolysis by Ran is extremely slow, and RanGAP is needed in the cytoplasm to stimulate the hydrolysis rate by several orders of magnitude (Bischoff et al., 1994; Klebe et al., 1995; Cook et al., 2007). However this enhancement is drastically dampened when RanGTP is associated with karyopherins (Floer and Blobel, 1996; Bischoff and Görlich, 1997; Fried and Kutay, 2003), and RanGAP-mediated stimulation of GTP-hydrolysis necessitates the implication of RanBPs, which destabilize the RanGTP/karyopherin complex. RanGTP hydrolysis therefore occurs at the cytoplasmic face of the NPC and involves the combined action of RanBPs and RanGAP (Figure 2, step 5). Upon GTP hydrolysis, Ran switches conformation, both at the level of the ‘switch I’ and ‘switch II’ regions, but also at the level of its C-terminal extension that folds as an α-helix and packs against the Ran structural core (Vetter et al., 1999; Scheffzek et al., 1995). Altogether, these structural rearrangements provide a steric barrier that prevents RanGDP from binding back to the karyopherin. After RanGTP release from Trn1 and GTP hydrolyis, Trn1 is again free in the cytoplasm and ready for another import cycle (Figure 2, step 1).

## 3 Nuclear Localization Signal Recognition by Transportin-1

In the years following the identification of Trn1 as the nuclear import receptor of hnRNP A1 (Pollard et al., 1996; Fridell et al., 1997; Bonifaci et al., 1997), several other proteins (including hnRNP D, hnRNP F, hnRNP M, HuR, Y-box binding protein 1, TAP, and histones H2A, H2B, H3 and H4) have been identified as Trn1 cargoes (Siomi et al., 1997; Fan and Steitz, 1998; Truant et al., 1999; Mühlhäusser et al., 2001; Rebane et al., 2004; Güttinger et al., 2004; Bader and Vogt, 2005; Suzuki et al., 2005). However, the lack of sequence similarity between the different NLSs has for a long time impeded the identification of the important elements defining NLSs recognized by Trn1. The understanding at a molecular level of the NLS recognition by Trn1 made a decisive step forward with the resolution of the first structure of Trn1 in complex with an NLS, namely the ‘M9’ NLS of hnRNP A1 (Figure 1C) (Lee et al., 2006). This work indeed provided the means to identify common patterns among seemingly contrasting NLSs and to group and classify them into a new class of NLSs called PY-NLSs. Following this pioneering work, several studies have refined the understanding of PY-NLS recognition by Trn1 (see subsection 3.1). However, many Trn1 cargoes harbour NLSs that do not resemble PY-NLSs (Chook and Süel, 2011; Twyffels et al., 2014). The recognition of these non-PY-NLSs by Trn1 is until now far less understood than that of PY-NLSs. Whether non-PY-NLSs can be regarded as variations around the PY-NLS paradigm, or whether they are too divergent and use completely different recognition rules, has not been clearly established yet, mostly by lack of studies, including structural ones, focused on non-PY-NLSs (see subsection 3.2). Finally, over the years, a salient aspect of Trn1-mediated nuclear import has emerged and consists in the many reported mechanisms that modulate the karyopherin-cargo interactions, thereby enabling nuclear import regulation (see subsection 3.3).

### 3.1 PY-NLS Recognition

Unlike the classical-NLSs recognized by the Impα/Impβ system, PY-NLSs cannot be described solely by a traditional consensus sequence. They are instead described by a collection of loose principles that can be summarized as follows: a peptide segment of 15–30 residues with intrinsic structural disorder, an overall basic character, and some weakly conserved sequence motifs, including a relatively conserved proline–tyrosine (PY) dipeptide, which gave the name PY-NLS to this class of signals (Lee et al., 2006). More precisely, the sequence motifs of PY-NLSs are composed of an N-terminal basic or hydrophobic motif followed by a C-terminal R/K/H-X_2–5_-P-Y motif (Lee et al., 2006). The nature of the N-terminal motif further divides PY-NLSs into basic and hydrophobic subclasses (bPY-NLSs and hPY-NLSs, respectively). In bPY-NLSs, the N-terminal motif consists of a patch of basic residues, whereas in hPY-NLSs, the equivalent N-terminal motif conforms to the loose Φ–G/A/S–Φ–Φ consensus (where Φ is a hydrophobic residue).

**FIGURE 3:**
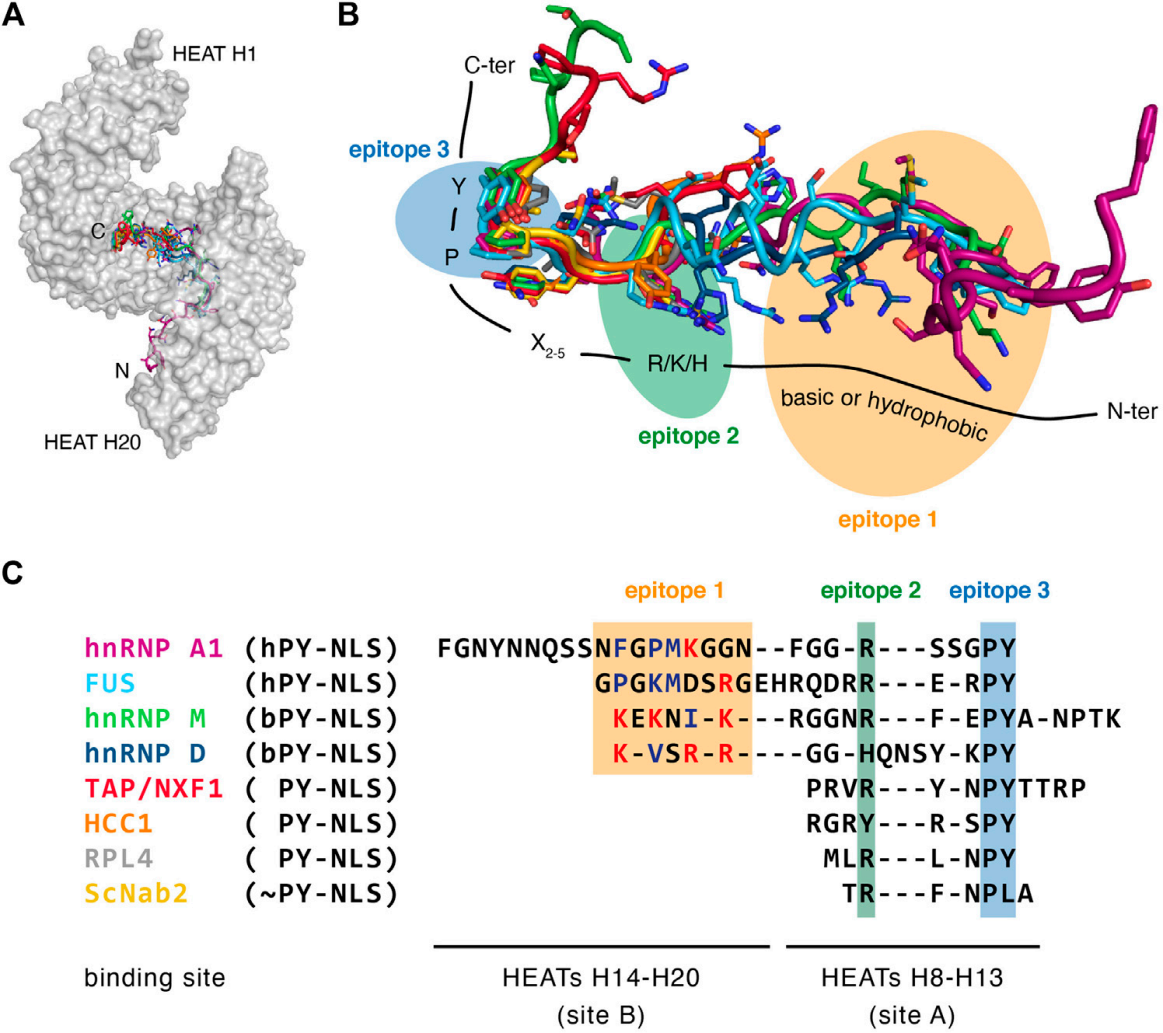
PY-NLS recognition by Transportin-1. **(A)** A common binding site for PY-NLSs with the C-terminal arch of Trn1. Trn1 is shown in gray as a transparent surface to facilitate PY-NLS observation. The PY-NLSs are displayed with different colors (see below), and all share the same N-ter to C-ter orientation. **(B)** Structural superposition of PY-NLSs from the following proteins: hnRNP A1 in purple [PDB code 2H4M (Lee et al., 2006)], FUS in light blue [PDB code 4FDD (Zhang and Chook, 2012)], hnRNP M in green [PDB code 2OT8 (Cansizoglu et al., 2007)], hnRNP D in dark blue [PDB code 2Z5N (Imasaki et al., 2007)], TAP/NXF1 in red [PDB code 2Z5K (Imasaki et al., 2007)], HCC1 in orange (PDB code 4OO6), RPL4 in gray [PDB code 5TQC (Huber and Hoelz, 2017)], ScNab2 in yellow [PDB code 4JLQ (Soniat et al., 2013)]. The structures were superimposed on the Trn1 proteins, but for clarity they are not shown, and only the PY-NLSs are displayed as sticks. The three recognition epitopes are shown with colored ellipses. Epitope 1, which corresponds to a stretch of basic or hydrophobic residues, is highlighted in yellow. Epitope 2, which corresponds to a positively charged residue (R/K/H), is highlighted in green. Epitope 3, which corresponds to a proline–tyrosine dipeptide (PY) is highlighted in blue. **(C)** Structure-based alignment of the same PY-NLSs displayed on panel B. The PY-NLS sequences were aligned according to their relative position in the structures. Only residues actually observed in the structures are displayed. The three epitopes as described in panel B are highlighted with the same color code. Within epitope 1, hydrophobic residues are colored in blue, and positively charged residues are colored in red. hPY-NLS: hydrophobic PY-NLS. bPY-NLS: basic PY-NLS. PY-NLS: PY-NLS without a basic or hydrophobic stretch seen to interact with Trn1 in the structure. ∼PY-NLS: variation to the PY-NLS motif.

To date, ∼10 distinct crystal structures of Trn1 have been solved in complex with PY-NLS substrates, including both bPY-NLSs and hPY-NLSs (Table 1). Collectively, these structures show that all PY-NLSs interact with the same region of Trn1, namely an extended zone of the concave face of the C-terminal arch (Figure 3A). However, considering the poor resemblance of the NLSs in term of sequence, it is not surprising to see that structural superposition of the different PY-NLSs is really far from perfect (Figure 3B). PY-NLSs vary both in length and sequence, and their binding mode to Trn1 may at first sight look very disparate. However, structural convergence clearly appears at two sites in the C-terminal part of the NLSs, and to a lesser extent at one site in the N-terminal part. These three sites of structural convergence form three binding epitopes, which have been initially identified by the structural comparison of the binding mode of hnRNP A1 and hnRNP M PY-NLSs (Cansizoglu et al., 2007), and were later shown to provide a significant energetic contribution to the binding (Süel et al., 2008). Epitope 1, which shows the least structural convergence and the highest sequence divergence, corresponds to the N-terminal motif presented above and can be formed by a patch of either basic or hydrophobic residues. Epitope 2 and 3 correspond to the positively charged residue and to the PY dipeptide of the C-terminal R/K/H-X_2–5_-P-Y motif, respectively (Figure 3B,C).

What emerges from such structural superposition, is that there are clearly two types of PY-NLSs: the ones that are seen to interact with Trn1 all along the three epitopes (e.g., hnRNP A1, FUS, hnRNP M and hnRNP D) and those that are only interacting at epitopes 2 and 3 (e.g., TAP/NXF1, HCC1, RPL4 and ScNab2). To help describe these two categories of PY-NLSs, and in connection with the structural heterogeneity and the segmental nature of Trn1 (Figure 1D), two main NLS-binding sites have been defined within the Trn1 C-terminal arch. The first one, called ‘site A’, corresponds to HEATs H8-H13, and represents the site of interaction of epitopes 2 and 3 (Figure 3C). The second one, called ‘site B’, corresponds to HEATs H14-H20, and is the site of interaction of epitope 1 (Figure 3C). PY-NLSs are thus interacting at both site A and site B, or uniquely at site A for the ones only interacting at the level of epitopes 2 and 3 (Imasaki et al., 2007). Since the conformation of site A is independent of NLS binding, whereas that of site B is intrinsically more adaptable, it has been proposed that the C-terminal R/K/H-X_2–5_-P-Y motif would bind first to site A, then a conformational change of site B would occur owing to a rearrangement at the level of the H13-H14 hinge, which with a sort of induced fit mechanism would adapt site B to epitope 1 binding (Imasaki et al., 2007). As previously mentioned, substrate dissociation upon RanGTP binding is likely occurring in the reverse order, with PY-NLSs dissociating first from site B, and then from site A (Imasaki et al., 2007). Molecular mechanisms governing proper functioning of Trn1 (e.g., substrate binding and release) are therefore deeply linked to its structural adaptability.

It is important to mention that although the structures of PY-NLSs in complex with Trn1 do not always show an interaction at the level of site B (Figures 3B,C), residues upstream of the R/K/H-X_2–5_-P-Y motif seen to interact at site A can significantly contribute to the overall binding (Imasaki et al., 2007; Süel et al., 2008). For that reason, PY-NLSs shown to only interact at site A may still be classified as bPY-NLSs or hPY-NLSs depending on their amino-acid composition (Lee et al., 2006; Twyffels et al., 2014). Most importantly, the binding site accommodating epitope 1 seems to be highly tolerant, since it can bind to both basic and hydrophobic patches, and a combination of both basic and hydrophobic residues is often observed in the N-terminal motif (Figure 3C) (Lee et al., 2006). Overall, the distribution of the binding energy along the three binding epitopes highly depends on the PY-NLS considered. In some cases, the contribution of epitope 3 is pre-eminent, whereas in others main binding contributions are provided by epitopes 1 and 2 (Süel et al., 2008; Lee et al., 2006; Imasaki et al., 2007; Cansizoglu et al., 2007). This property, together with the fact that the epitopes are energetically quasi-independent, had been proposed as responsible for the high adaptability of the epitopes, which can accommodate large sequence diversity on condition that the others are energetically strong. A combinatorial mixing of energetically weak and strong motifs indeed maintains an overall affinity compatible with nuclear import (Süel et al., 2008).

### 3.2 Non-PY-NLS Recognition

In addition to the import of PY-NLS-containing cargoes, Trn1 is implicated in the import of various cargoes obviously lacking a PY-NLS (Twyffels et al., 2014; Chook and Süel, 2011). These cargoes are imported by Trn1 via non-PY-NLSs, and include diverse proteins such as the core histones (H2A, H2B, H3 and H4) (Mühlhäusser et al., 2001; Baake et al., 2001; Mosammaparast et al., 2002; Blackwell et al., 2007; Soniat et al., 2016), ribosomal proteins (RPL23A, RPL5, RPL7 and RPS7) (Jäkel and Görlich, 1998; Tai et al., 2013), viral proteins (Arnold et al., 2006; Le Roux and Moroianu, 2003; Klucevsek et al., 2006), the RNA-editing enzyme ADAR1 (Fritz et al., 2009; Barraud et al., 2014; Banerjee and Barraud, 2014), the transcription factor FOXO4 (Putker et al., 2013), and the cold-inducible RNA-binding protein CIRBP (Bourgeois et al., 2020). With some exceptions, PY-NLS seem to be specific to Trn1, while non-PY-NLS can be imported by Trn1 but can frequently be recognized and imported by multiple karyopherins such as the heterodimer Impα/Impβ (Kimura et al., 2013; Twyffels et al., 2014).

Structural understanding of non-PY-NLS recognition by Trn1 is quite limited, since only a single structure of Trn1 in complex with a non-PY-NLS peptide has been solved to date (Soniat and Chook, 2016). In this structure, the non-PY-NLS of histone H3 spans HEATs H11-H18 in the concave site of the Trn1 C-terminal arch. It occupies similar positions as the ones of PY-NLSs bound to Trn1, with the exception that there is no PY dipeptide or other residues occupying the epitope 3 recognition site of PY-NLSs (Figure 3). A positively charged stretch binds the site for PY-NLS epitope 1, and an arginine residue occupies the PY-NLS epitope 2 position (Soniat and Chook, 2016). The binding mode is therefore somewhat similar to the one of PY-NLSs, but uses a strong epitope 1 that compensates for the loss of the PY motif. The non-PY-NLS of histone H3 can thus be seen as an ultimate variation of the PY-NLS, lacking the PY motif, but retaining the other hallmarks of this class of NLSs. Whether this aspect is unique to histone H3, or is shared by other non-PY-NLSs of Trn1, is a likely possibility, but remains to be determined by further studies, including structural works dealing with other non-PY-NLSs.

Although no other crystal structure of non-PY-NLSs with Trn1 has been obtained, molecular information on non-PY-NLS interactions with Trn1 are available for several systems. These include the FOXO4 (forkhead box O4) protein, which binds Trn1 using a very unusual mechanism (Putker et al., 2013). Upon accumulation of reactive oxidative species (ROS), a disulfide bond is formed between a cysteine residue of FOXO4 and a cysteine residue in Trn1. The formation of a covalent complex ensures a strong interaction with Trn1 and an efficient nuclear import. In the nucleus, the more reducing environment facilitates the reduction of the intermolecular disulfide bond and the release of the FOXO4 transcription factor, which eventually activates transcription of ROS-detoxifying enzymes. Neither the interaction site in Trn1, nor the implicated cysteine, have so far been identified. Residues surrounding the implicated cysteine in FOXO4 have been shown to participate in the binding to Trn1 (Putker et al., 2013), but it remains to be determined whether this interaction occurs specifically at a single site on Trn1 surface or at several positions. Among other things, it would be interesting to know whether the present binding site is completely different from the one used by PY-NLSs or if they have some parts in common.

A non-PY-NLS for which molecular details of its recognition by Trn1 is available consists of the atypical NLS of the RNA editing enzyme ADAR1 (Eckmann et al., 2001; Strehblow et al., 2002; Fritz et al., 2009). This NLS overlaps the third double-stranded RNA-binding domain (dsRBD) of the protein (Barraud and Allain, 2012), and shows no similarity to PY-NLSs. The molecular basis for the dsRBD-mediated nuclear import of ADAR1 was investigated at a molecular level. The solution structure of the ADAR1-dsRBD3 revealed an extended dsRBD fold with an additional α-helix in the N-terminus. This extension radically changes the relative position of the flexible fragments flanking the dsRBD and brings the N- and C-terminal flanking regions in close proximity (Barraud et al., 2014). The two fragments flanking the folded dsRBD were shown to constitute two essential modules involved in the interaction with Trn1 and the non-PY-NLS of ADAR1 was thus called ‘bimodular NLS’. The intervening dsRBD was shown to only act as a scaffolding domain, which properly positions the N- and C-terminal modules for an effective interaction with Trn1. Functional bimodular NLSs could indeed be designed by replacing the ADAR1-dsRBD3 with an unrelated dsRBD, or even with a small peptide linker, which clearly indicates that the dsRBD only helps bring together the two NLS-modules that are otherwise distantly spaced in the protein sequence (Barraud et al., 2014). Molecular modelling and functional assays involving Trn1 mutants affected in the regular PY-NLS binding sites (Figure 3), namely ‘site A’ and ‘site B’, suggested that the bimodular NLS of ADAR1 may interact with Trn1 at the same interaction sites as the ones of PY-NLSs. The folded dsRBD would be small enough to insert into the C-terminal arch of Trn1, and the N- and C-terminal modules of the non-PY-NLS of ADAR1 could adopt an extended conformation that allows interaction with Trn1 at the position of epitopes 1–3, even though they do not contain a PY dipeptide (Barraud et al., 2014). However, the atomic details of the interaction still remain to be uncovered, which would expand our understanding of the repertoire of non-PY-NLS recognition by Trn1.

In another recent report, a non-PY-NLS has been identified in the cold-inducible RNA-binding protein CIRBP and was shown to participate in the Trn1-mediated nuclear import of CIRBP (Bourgeois et al., 2020). This non-PY-NLS corresponds to a ∼40-residue region rich in RG and RGG motifs, called the RG/RGG region. This RG/RGG region binds to Trn1 in a RanGTP-competitive manner and does not contain PY or PΦ motifs (Φ: hydrophobic residue) that may be reminiscent of PY-NLSs. The most striking point regarding the RG/RGG region interaction with Trn1 consists in the involvement of the Trn1 H8 loop in the interaction (Bourgeois et al., 2020). The RG/RGG region of CIRBP was indeed shown to contact Trn1 at two key sites, a site competing with the binding of the PY-NLS of FUS, and a non-overlapping site within the unstructured H8 loop of Trn1. The importance of this additional site is reflected in the fact that deletion of the H8 loop reduces the binding affinity of the RG/RGG region to Trn1 by ∼5 fold (Bourgeois et al., 2020). As presented above, although the H8 loop has been shown to be essential for cargo release upon RanGTP binding, it was reported as dispensable for NLS binding, and was indeed deleted in most structures of Trn1 in complex with PY-NLSs (Table 1). This study concerning the RG/RGG NLS of CIRBP raises the possibility that the Trn1 H8 loop might be important for NLS binding, at least for certain non-PY-NLSs (Bourgeois et al., 2020). To determine whether the disordered H8 loop is important for the recognition of other non-PY-NLSs is definitely a critical question that would necessitate further studies.

### 3.3 Regulation of Trn1-Cargo Interactions

Several mechanisms modulating Trn1-cargo interactions, thereby enabling nuclear import regulation, have been reported in the literature. This mostly includes the modulation of Trn1-cargo interactions via post-translational modifications (e.g., arginine methylation, lysine acetylation, and serine or tyrosine phosphorylation), but also the enhancement or inhibition of the interaction by more elaborated mechanisms.

#### 3.1.1 Post-Translational Modifications Regulating Trn1-Cargo Interactions

Arginine methylation has been reported as a modulator of Trn1-cargo interactions in several contexts. First, asymmetric arginine dimethylation within the PY-NLS of the nuclear Polyadenylate-binding protein 2 (PABP-2) reduces its affinity for Trn1 (Fronz et al., 2011). Six arginine residues that are likely involved in Trn1-binding as part of PY-NLS epitopes 1 and 2, are subjected to post-translational modifications, which would explain the reduction of interaction with Trn1. It is worth noting that arginine methylation is not the only mechanism that modulates interaction of PABP-2 with Trn1, since binding of PABP-2 to poly-A RNA competes with binding to Trn1. The two mechanisms are interconnected since arginine methylation slightly enhances binding of PABP-2 to RNA, which altogether reduces its interaction with Trn1 (Fronz et al., 2011). In cells, whether this competition occurs in the cytoplasm or in the nucleus has not yet been firmly established. Similarly, asymmetric arginine dimethylation of FUS was reported to reduce interaction with Trn1 thereby affecting nuclear import (Dormann et al., 2012). In contrast to the PABP-2 situation, arginine methylation does not occur directly within the PY-NLS of FUS but next to it, in the so-called RGG3 region present just upstream of the C-terminal PY-NLS. Interestingly, although arginine dimethylation modulates Trn1 binding to wild-type FUS with a fully functional PY-NLS, the effect of arginine methylation in the RGG3 region is more pronounced in the case of FUS mutants with defective PY-NLSs, and inhibition of arginine methylation in these mutant proteins restores an efficient nuclear import (Dormann et al., 2012). Strikingly, it was later reported that in contrast to arginine dimethylation that drastically reduces the interaction of the RGG3 region of FUS with Trn1, arginine monomethylation only slightly affects this interaction and behaves similarly to unmethylated arginine in FUS (Suárez-Calvet et al., 2016). Owing to their almost identical domain organization, the FET proteins, namely FUS, EWS and TAF15, are similarly affected by arginine methylation in their interaction properties with Trn1 (Dormann et al., 2012; Suárez-Calvet et al., 2016). Along the same lines, arginine methylation of the non-PY-NLS of CIRBP, also referred to as the RG/RGG region, reduces its binding affinity to Trn1, thereby regulating nuclear import of CIRBP (Bourgeois et al., 2020).

Lysine acetylation has been reported to regulate the interaction of FUS with Trn1. A lysine acetylation within the PY-NLS of FUS was shown to disrupt the interaction between FUS and Trn1, resulting in the reduction of FUS nuclear import and hence its mislocalization in the cytoplasm (Arenas et al., 2020). The implicated lysine is situated in epitope 1 of FUS PY-NLS (Figure 3), and is directly in contact with a glutamate and an aspartate residue of Trn1 HEATs H14 and H15 (Zhang and Chook, 2012; Niu et al., 2012). The reduction of interaction with Trn1 upon acetylation of this specific lysine is thereby clearly in accordance with structural data.

Phosphorylations have been reported as modulator of Trn1-cargo interactions in several contexts. First, the Trn1-mediated import of Sam68 was reported as regulated through phosphorylation (Lukong et al., 2005). The BRK kinase indeed phosphorylates Sam68 on three tyrosine residues in its PY-NLS, including Y440 that forms the PY motif of PY-NLS epitope 3 (Figure 3). While unphosphorylated Sam68 is predominantly nuclear, Sam68 phosphorylated on Y440 relocalizes to cytoplasmic perinuclear structures (Lukong et al., 2005), most probably as a consequence of an impaired interaction with Trn1. Similarly, the FET proteins FUS and EWS have been shown to be phosphorylated on their C-terminal tyrosine (i.e., Y526 and Y656, respectively), which forms the PY motif of their PY-NLSs (Leemann-Zakaryan et al., 2011; Darovic et al., 2015). In the case of FUS, this phosphorylation completely abolishes the PY-NLS interaction with Trn1 (Darovic et al., 2015). The tyrosine residue that is phosphorylated forms epitope 3 of FUS PY-NLS (Figure 3), and contacts several Trn1 residues, including an aspartate residue in HEAT H8 (Zhang and Chook, 2012; Niu et al., 2012). The reduction of interaction with Trn1 upon phosphorylation is thereby clearly in accordance with structural data, since phosphorylation would cause steric hindrance and electrostatic repulsion between the PY motif and its binding site on Trn1 (Darovic et al., 2015). Finally, modulation of Trn1-cargo interaction upon phosphorylation has also been reported in the case of hnRNP A1 (Allemand et al., 2005). Upon osmotic stress, hnRNP A1 is relocalized from the nucleus to the cytoplasm. This cytoplasmic relocalization is dependent on the phosphorylation of several serine residues present at the C-terminus next to the ‘M9’ PY-NLS of hnRNP A1 (Allemand et al., 2005). Importantly, the hyperphosphorylation of hnRNP A1 C-terminal region reduces its ability to interact with Trn1. The mechanism by which hyperphosphorylation of serines near the PY-NLS of hnRNP A1 leads to impaired binding to Trn1 is unclear, but it has been proposed that these phosphorylations might modulate the accessibility of the PY-NLS (Allemand et al., 2005).

Interestingly, in a completely different context, osmotic stress also caused the cytoplasmic translocation of FUS in neurons (Hock et al., 2018). In this case, relocalization of FUS upon osmotic stress does not depend on phosphorylations of the cargo, but is caused by a global impairment of Trn1-mediated nuclear import. As a consequence, several Trn1 cargoes (e.g., EWS, TAF15, and hnRNP A1) also displayed a marked cytoplasmic shift in neurons (Hock et al., 2018).

#### 3.1.2 Reactive Oxidative Species Regulating Trn1-Cargo Interactions

As already introduced above with the example of FOXO4, reactive oxidative species (ROS) can modulate Trn1-cargo interaction, and thereby regulate nuclear import (Putker et al., 2013). In this example, accumulation of ROS leads to the formation of a covalent complex between FOXO4 NLS and Trn1, which ensures a strong Trn1-cargo interaction and an efficient nuclear import (Putker et al., 2013). In another example, ROS also stimulates the interaction of Trn1 with the Parkinson protein 7 (PARK7/DJ-1), but the mechanism seems very different (Björkblom et al., 2014). In this system, H_2_O_2_ treatment disrupts the DJ-1 dimeric complex, which renders the otherwise masked PY-NLS accessible for Trn1 binding, thereby activating nuclear import of monomeric DJ-1. Here ROS enhances the binding of the DJ-1 PY-NLS to Trn1, but the effect is likely indirect and triggered by DJ-1 monomerization (Björkblom et al., 2014). In a last example, it was also shown that ROS stimulates the interaction of Trn1 with the circadian clock protein PERIOD 1 (PER1) (Korge et al., 2018). Here, H_2_O_2_ treatment resulted in a dose-dependent increase of Trn1-PER1 interaction. It is worth mentioning that Trn1-binding to PER1 is abolished under reducing conditions, which suggests that a disulfide bond might be formed between a cysteine residue of PER1 and a cysteine residue in Trn1, as reported for FOXO4 (Putker et al., 2013). Unexpectedly, increased binding upon H_2_O_2_ treatment did not lead to an increased nuclear import of PER1, but was rather associated with a specific slow-down of PER1 nuclear import (Korge et al., 2018). This intriguing observation might be related to the fact that PER1 is translocated into the nucleus by several karyopherins, but would definitely deserve further examination.

#### 3.1.3 Masking/Unmasking Functional NLS By Folding/Unfolding Transitions

In an intriguing example, modulation of Trn1-cargo interaction would involve the folding/unfolding of a zinc-finger (ZnF). In the TIS11 family of proteins, a cryptic PY-NLS is masked within a small ZnF domain, the folding of which competes with Trn1-mediated nuclear import (Twyffels et al., 2013). It is proposed that TIS11 proteins could be in equilibrium between two states, one in which the ZnF is firmly folded, and one in which it is partially unfolded. This latter state would allow the cryptic PY-NLS to become accessible and thus to mediate nuclear translocation of the protein (Twyffels et al., 2013).

#### 3.1.4 RNA-Mediated Regulation of Trn1-Cargo Interactions

Another example of regulation of nuclear import by Trn1 consists in the RNA-mediated regulation of ADAR1 import. As presented above, the non-PY-NLS of ADAR1 is bimodular and formed by the N- and C-terminal extensions flanking a dsRBD. This molecular organization was shown to act as an RNA-sensing NLS that can be switched on and off, depending on the presence of dsRNA associated with the dsRBD (Fritz et al., 2009; Barraud et al., 2014). The non-PY-NLS of ADAR1 presents two functional and non-overlapping interaction surfaces, namely a functional dsRNA-binding interface involving the central dsRBD, and a functional Trn1-binding interface that consists of the N- and C-terminal modules flanking the folded domain. Even if these two interfaces are distinct, the non-PY-NLS of ADAR1 cannot bind to dsRNA and Trn1 simultaneously, most probably for steric reasons, which explains how this non-PY-NLS functions as an RNA-sensing NLS (Barraud et al., 2014). Interestingly, RNA-binding enhances nuclear export of ADAR1 (Fritz et al., 2009), which suggests that ADAR1 might leave the nucleus bound to substrate RNAs. The binding of the non-PY-NLS to Trn1 in the cytoplasm could then help the dissociation of ADAR1-bound RNAs and the RNA-sensing NLS would then prevent ADAR1 from carrying RNAs back into the nucleus (Barraud et al., 2014).

In another system, RNA has been shown not to impair, but to stimulate Trn1-cargo interaction. A circular RNA named circAnks1a, was indeed shown to enhance the interaction between the transcription factor YB-1 and Trn1, thus promoting the Trn1-mediated translocation of YB-1 into the nucleus (Zhang et al., 2019). The mechanism enabling the PY-NLS of YB-1 (Mordovkina et al., 2016) to interact more strongly with Trn1 in presence of RNA has not yet been investigated and would deserve further studies. Altogether, these examples illustrate the multiple layers and the different mechanisms that can affect Trn1-cargo interaction and regulate nuclear import.

## 4 Moonlighting Functions of Transportin-1

**FIGURE 4:**
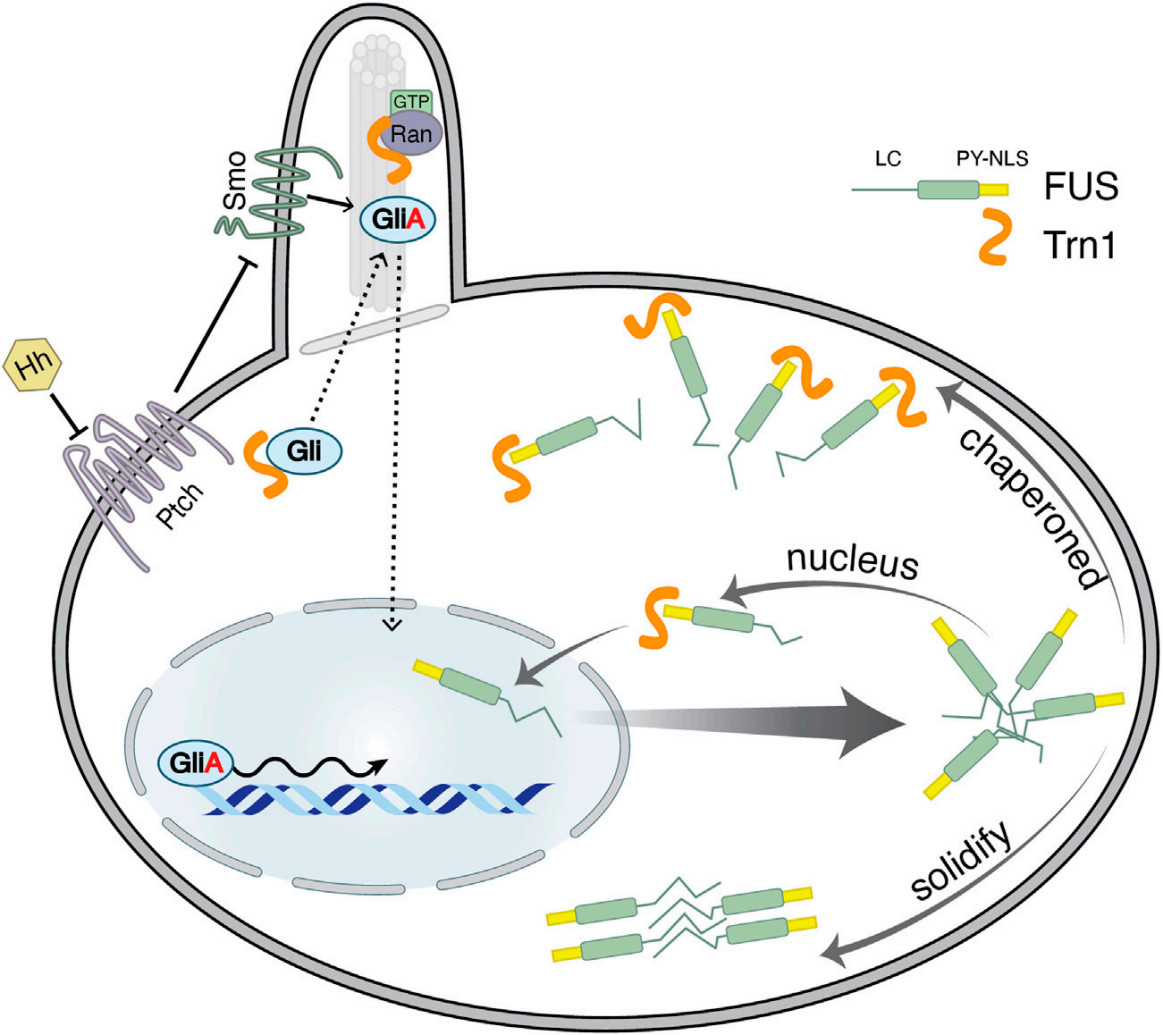
Moonlighting functions of Transportin-1. On one hand, Trn1 has been caught in trafficking proteins destined to cilia (Dishinger et al., 2010; Hurd et al., 2011). For instance, Gli, a transcription factor associated with Hedgehog (Hh) signalling is shown to interact with Trn1 via its ciliary localization signal (CLS) (Han et al., 2017). Ran-GTP in the cilia appears to release Gli from Trn1. Upon Hh signalling Gli is converted to an activated form Gli^A^, a transcriptional activator that then translocates to the nucleus to drive transcription (Kim et al., 2009; Han et al., 2017). On the other hand, Trn1 regulates the aggregation of mislocalized RNA-binding proteins (RBPs) in the cytoplasm (Guo et al., 2018; Hock et al., 2018; Hofweber et al., 2018; Qamar et al., 2018; Yoshizawa et al., 2018). Upon stress or when mutated, FUS translocates into the cytoplasm, where it can aggregate and further solidify into pathologic foci. Trn1 recognizes PY-NLS of FUS and directly drives it back into the nucleus (Dormann et al., 2010). Moreover, Trn1 can additionally bind to the low-complexity (LC) and folded region of FUS. This binding competes with FUS–FUS interaction and protein-assembly. Thus, PY-NLS serves as a cytoplasmic signal such that mislocalized nuclear RBPs are specifically chaperoned by Trn1 (Guo et al., 2018; Hock et al., 2018; Hofweber et al., 2018; Qamar et al., 2018; Yoshizawa et al., 2018).

Besides nuclear trafficking, Trn1 has been shown to conduct a larger symphony of various cellular processes. These include ciliary transport, stress granule formation, and virus uncoating (Figure 4). In this section, we briefly describe the role of Trn1 in these processes.

### 4.1 Ciliary Transport

Cilia are protrusions on the cell surface, which help to bridge cells to the external environment. Primary cilia are frequently used for cell motility but can also play a key role in both chemo- and mechano-sensing. Further, several signalling pathways depend on proper ciliary function. Disruption of ciliary function leads to ciliopathies, an emerging disease condition (Reiter and Leroux, 2017). Until recently, a clear picture of proteins destined to ciliary targeting remained obscure. Meanwhile, the similarity between nuclear and ciliary transport has been demonstrated for several cases where cargo proteins interact with nuclear receptors to be transported to cilia. In addition to a classical NLS-like ciliary localization signal (CLS), which depends upon Importin-β, CLSs have been identified that bind Trn1 to mediate their ciliary targeting (Fan et al., 2007; Dishinger et al., 2010; Hurd et al., 2011).

Retinitis pigmentosa is an X-linked disorder causing night blindness that eventually leads to permanent blindness (Patil et al., 2011). Mutations in the protein Retinitis pigmentosa 2 (RP2) are responsible for the disorder. Trn1-mediated transport of RP2 to cilia has been demonstrated (Hurd et al., 2011). The interaction between RP2 and Trn1 is mediated by two distinct sites that bind Trn1 independently. The first binding site is located at the N-terminus of RP2 and is similar to an NLS. The other binding site overlaps with the tubulin folding cofactor C (TBCC) domain of RP2 and shows slight similarity to the ‘M9’ sequence of hnRNP A1. Although both of these sites interact with Trn1 independently, only the M9-like sequence is essential for RP2 ciliary targeting (Hurd et al., 2011). Besides, mutations of the M9-like sequence were shown to abolish ciliary targeting of RP2 and were found mutated in several human diseases (Patil et al., 2011). Localization of RP2 to the plasma membrane was shown to be a prerequisite for RP2 and Trn1 interaction. The plasma membrane association of RP2 is a direct consequence of myristoylation and palmitoylation of residues Gly2 and Cys3.

Primary cilia harbour many factors required for Hedgehog (Hh) signalling (Briscoe and Thérond, 2013). Hh signalling plays a central role in development and tissue homoeostasis. Mis-regulation of the Hh signalling pathway results in developmental disorders and cancers. Trn1 has been shown to regulate Hh signalling by trafficking Gli transcription factor into cilia, where upon Hh signalling Gli is converted to activate the Gli^A^ transcriptional activator that then translocates to the nucleus to drive transcription (Figure 4) (Kim et al., 2009; Han et al., 2017). Interestingly, Trn1 itself is transcriptionally activated by Gli^A^ (Niewiadomski et al., 2014). Gli proteins exhibit both classical NLS and PY-NLS. The PY-like NLS along with the C-terminal residues (aa 874–1,080) is vital for the ciliary localization while the classical NLS is responsible for nuclear localization (Han et al., 2017). A knockdown of Trn1 diminishes ciliary localization of Gli both in the presence and in the absence of Hg signalling. Further, ciliary localization of Gli is reverted on transfecting Trn1 highlighting that Trn1 is necessary for Gli ciliary localization (Han et al., 2017). In addition, it was shown that Trn1 is required for Hh signalling and is therefore critical for zebrafish embryonic development.

Intra-flagellar transport (IFT) is mainly propelled by Kinesin-2 family members (Dishinger et al., 2010; Verhey et al., 2011). KIF17 is a member of the Kinesin-2 family. The similarity between Trn1-mediated nuclear and ciliary trafficking had been initially suggested from the conservation of common features (Devos et al., 2004), and direct evidence were reported few years later (Dishinger et al., 2010). It has been shown that Trn1 and RanGTP govern ciliary entry of KIF17 (Dishinger et al., 2010). Indeed, the authors have identified NLS-like sequences in KIF17: amino acids 767–777 (KRRKR) and 1,016–1,019 (KRKK). Only mutations in 1,016–1,019 (CLS) of the KIF17 tail domain abolished ciliary targeting and interaction with Trn1, whereas mutations in aa 767–772 did not affect ciliary entry. These findings indicate that Trn1 indeed governs the trafficking of KIF17 to cilia. Moreover, the C-terminal tail domain (amino acids 801–1,028) when fused to a non-ciliary kinesin promotes localization to cilia, indicating that this CLS is necessary and sufficient for ciliary transport. Consistently, a mutated CLS fused to non-ciliary kinesin did not promote transport into cilia. Further, the presence of RanGTP in cilia has been demonstrated using RanGTP specific antibodies (Fan et al., 2011). Also mass spectrometric analyses have documented the presence of Trn1 and RanGTP in the cilium (Liu et al., 2007; Ishikawa et al., 2012; Narita et al., 2012).

### 4.2 Virus Uncoating

Understanding the molecular mechanisms of host-virus interactions is of great medical interest. Virus uncoating is one of the critical steps in virus infection which involves the timely release of the viral genome from its shell/capsid (Yamauchi and Greber, 2016; Helenius, 2018). The stimulus and mechanistic details of virus uncoating remain largely concealed. Virus infection in eukaryotic cells often depends on the nuclear transport machinery (Cohen et al., 2011; Matreyek and Engelman, 2013; Tessier et al., 2019). Recent studies have shown a direct role of Trn1 in virus uncoating (Fernandez et al., 2019; Miyake et al., 2019; Carlon-Andres et al., 2020; Yamauchi, 2020).

Uncoating of Influenza virus A (IVA) happens in three critical stages: priming, M1 shell dissociation and virus ribonucleoprotein untangling. Trn1 was found among the novel hits in short interfering RNA (siRNA)-based infection screenings to identify host proteins associated with viral cytosolic uncoating and nuclear import (Miyake et al., 2019). Upon Trn1 depletion by siRNA or short hairpin RNA (shRNA) knockdown, a drastic reduction in the infection rate (66–79%) in different cell types was observed. Infection was reversed upon expression of GFP-Trn1 that was insensitive to si or sh RNA. Immunofluorescence performed 4 hours post infection demonstrated the majority of the M1-ribonucleoprotein (vRNP) to be present in the cytosol in Trn1 depleted cells while M1-vRNP was present in the nucleus in MOCK depleted cells (Miyake et al., 2019). Glycine 18 and adjacent residues of M1 was identified to be essential for an interaction with Trn1. Further, a G18A mutation in M1 was shown to affect both viral assembly and uncoating, ultimately resulting in a drastic reduction in infection (Yamauchi, 2020). The atomic structure of G18A showed no major rearrangement when compared to wild-type M1, indicating that the mutation most likely affects the interaction with Trn1.

Capsid protein (CA) multimers encase the RNA genome of human immunodeficiency virus-1 (HIV-1) (Campbell and Hope, 2015). The capsid is believed to provide a secured micro-environment for the viral reverse transcriptase to transform RNA genome to double-stranded DNA and to evade the cell’s innate immune system (Lahaye et al., 2013; Rasaiyaah et al., 2013). It is largely accepted that timely dissolution of CA is mandatory for efficient infection but the exact mechanism remains unclear. Since HIV-1 uncoating is linked to nuclear entry, Trn1 seems to play a critical role in HIV-1 infection (Fernandez et al., 2019). To test this, the authors knocked down Trn1, which led to a significant reduction in HIV-1 infection. While Trn1 transfection restored HIV-1 infection, implying a direct role of Trn1 in HIV-1 infection. Although Trn1 and Trn2 share 83% identity, Trn2 knockdown did not affect HIV-1 infection, suggesting a specific function of Trn1. Moreover, a significant reduction in infection was seen in Trn1 depleted CD4^+^ cells, substantiating that Trn1 is a prerequisite for early steps of infection (Fernandez et al., 2019). CA and Trn1 could be co-immunoprecipitated in HIV-1- infected cells, indicating interaction of Trn1 with CA. Further, Trn1 colocalization with CA in the cytosol and at the nuclear pore was shown. CA exhibits a hydrophobic patch containing a glycine residue at position 89 (Fernandez et al., 2019). Such a glycine residue was shown to be critical for interaction of hnRNP A1 PY-NLS with Trn1 (Lee et al., 2006). Consistently, the glycine at position 89 (G89) is conserved throughout HIV-1 subtypes (Kuiken et al., 2003) and G89 in conjunction with the proline at position 90 (P90) was shown to bind the peptidylprolyl isomerase CypA (Gamble et al., 1996; Yoo et al., 1997). A G89V mutation drastically reduced the infection rate when compared to a P90A mutation, suggesting a dependency of G89 for the interaction with Trn1. Further, the dependency of Trn1-CA interaction on G89 was shown *in vitro* by surface plasmon resonance (Fernandez et al., 2019). The authors demonstrated the HIV-1 CA uncoating dependency on Trn1 by an elegant fate-of-capsid assay on sedimentation gradients. Soluble CA, representing fully uncoated capsid was only present in control HeLa cytosolic fractions and not in Trn1 knockdown lysates. Purified recombinant Trn1 was also shown to cause structural damage to the assembled capsid/nucleocapsid (CANC) by atomic force microscopy. Additionally, W730 of Trn1 was shown to be critical for the interaction with CA and uncoating (Fernandez et al., 2019). Molecular docking simulations revealed that Trn1 may have other points of contact in addition to G89 with CA hexamers and it may induce strong hindrance leading to uncoating. Moreover, Trn1 depletion significantly reduced nuclear accumulation of both CA and viral DNA. While CA and viral pre-integration complex DNA (PIC DNA) accumulated in the nucleus on Trn1 expression, suggesting that Trn1 traffics both capsid and PIC DNA.

### 4.3 Regulation of Stress Granule Formation

As mentioned previously, nuclear import of numerous RNA-binding proteins (RBPs) is controlled by Trn1, including the FET protein family (FUS, EWS, TAF15) and hnRNP A1/A2 (Lee et al., 2006). Upon stress response or when mutated, these predominantly nuclear proteins can translocate to the cytoplasm and accumulate in stress granules (SGs). SGs can further mature into pathogenic inclusions that are typical for a group of fatal neurodegenerative disorders including amyotrophic lateral sclerosis (ALS), frontotemporal dementia (FTD), and multisystem proteinopathy (MSP) (Dormann et al., 2010; Harrison and Shorter, 2017; Neumann et al., 2011). Recent studies show, that Trn1 not only acts as a transport receptor but presumably serves as a powerful cytoplasmic chaperone for mislocalized RBPs. Thus, Trn1 regulates SGs formation and their further fibrillization (Figure 4) (Guo et al., 2018; Hock et al., 2018; Hofweber et al., 2018; Qamar et al., 2018; Yoshizawa et al., 2018; Sun et al., 2020).

#### 4.1.1 Trn1 is a Chaperone of Mislocalized FET-Proteins

FET proteins are involved in many steps of RNA metabolism such as transcription and splicing (Lagier-Tourenne et al., 2010). Moreover, they possess additional cytoplasmic function e.g., FUS modulates the axonal mRNA transport and local translation (Fujii and Takumi, 2005; López-Erauskin et al., 2020). FET proteins share a common domain-architecture: An N-terminal Prion-like (PrLD) or low complexity domain (LC), followed by several arginine-glycine-glycine (RGG) regions, an RNA recognition motif (RRM) and a C-terminal PY-NLS (Iko et al., 2004; Springhower et al., 2020).

ALS-associated FUS mutations are usually found in the PY-NLS and directly disturb nuclear import mediated by Trn1. This leads to FUS cytoplasmic retention and subsequent disease manifestation (Dormann et al., 2010). However, recent studies showed an additional chaperoning function of Trn1 that shapes phase separation of FUS and other FET proteins. The PY-NLS anchors Trn1 to mislocalized cargos and promotes additional weak binding to low complexity and folded regions which compete with self-aggregation of FET proteins (Figure 4) (Guo et al., 2018; Hofweber et al., 2018; Qamar et al., 2018; Yoshizawa et al., 2018). This chaperoning function seems to be independent of Trn1-mediated nuclear import, as import mutants of Trn1 (Chook et al., 2002) still prevent and reverse FUS fibrillization. In addition, Trn1 can specifically extract its cargos from SGs without affecting SG-formation per se (Guo et al., 2018). Thus, in this case, the NLS might serve as a signal that ensures that nuclear cargos are chaperoned and disaggregated when they are trapped in the cytoplasm (Figure 4) (Guo et al., 2018; Hofweber et al., 2018; Qamar et al., 2018; Yoshizawa et al., 2018). Similar mechanisms have been proposed for the Trn1-mediated disaggregation of the amyloid fibrils formed by hnRNP A1 LC domain, thus conferring a protective activity against hnRNP A1-driven ALS and MSP (Sun et al., 2020).

Chaperoning is presumably a common feature of importins, given that Impα/Impβ could prevent TDP43 fibrillization *in vitro* (Guo et al., 2018). Moreover, Impα/Impβ inhibits phase separation of FUS if fused to a classical NLS (Yoshizawa et al., 2018). Importin-mediated disaggregation might be similar to the transition of the nuclear pore complex, where importins break weak hydrophobic interactions between FG-Nups (Schmidt and Görlich, 2016). Corresponding to the interaction with nucleoporins, Trn1 interacts with tyrosines in the FUS low-complexity domain. Moreover, Trn1 contains highly acidic surfaces and loops that possibly interact with the basic RGG regions of FUS (Yoshizawa et al., 2018). Thus, Trn1 and presumably importins in general, might serve as cytoplasmic chaperones, that regulate the dynamics of phase separation and thereby the content of stress granules.

#### 4.1.2 Chaperoning Modulation By Arginine Methylation and RNA-Binding

Arginine methylation can modulate not only direct recognition and binding to FUS-NLS as mentioned before (Dormann et al., 2012), but also chaperoning of FUS by Trn1. FUS-FTD is usually not linked to FUS mutations although its manifestation is analogous to ALS. Immunohistochemistry, however, revealed that the FUS-FTD inclusions contain hypomethylated arginines, hence suggesting a different pathomolecular mechanism that leads to fibril formation in FUS-FTDs (Dormann et al., 2012). Cation-π interaction between hypomethylated arginines in RGG and tyrosines in LC promotes FUS condensation into stable inter-molecular β-sheet-rich hydrogels, that cannot be disassociated by physiological Trn1-level (Qamar et al., 2018). Thus, methylation of arginines in RGG regions tunes the strength of FUS intermolecular interactions that promote phase separation (Hofweber et al., 2018; Qamar et al., 2018).

In the nucleus, high concentration of RNA saturates binding properties of FUS molecules and inhibits phase separation. In contrary, the low RNA concentration in the cytoplasm may promote FUS-RNP assembly (Maharana et al., 2018). RGG regions bind RNA and Trn1 in a mutually exclusive manner. Thus, RNA replacement by Trn1 might also contribute to FUS chaperoning (Hofweber et al., 2018; Yoshizawa et al., 2018). ALS-associated arginine-mutations lead to aberrant RNA binding that is static and, on the contrary to wild-type FUS, does not switch into dynamic binding stage and promotes larger droplets formation (Niaki et al., 2020).

## 5 Concluding Remarks

The recognition at a molecular level of NLSs by Trn1 is now relatively well understood at least for the PY-NLS family. The recognition of non-PY-NLSs by Trn1 is however far less understood and would definitely deserve comprehensive biochemical, structural and cellular studies. These studies focussed on non-PY-NLS recognition by Trn1 would be pivotal to achieve a broad understanding of the mode of action of Trn1 and to shed light on this rather unexplored area of Trn1-cargo recognition. It is worth noting that in the study of the CIRBP non-PY-NLS, an unexpected role for the H8 loop in NLS binding has been unveiled (Bourgeois et al., 2020). The role of the H8 loop has been traditionally restricted to cargo dissociation upon RanGTP binding, and was reported as dispensable for NLS binding in early studies (Lee et al., 2006; Imasaki et al., 2007). This new study thereby raises the possibility that the Trn1 H8 loop might also be important for binding to particular NLSs.

Although the implication of Trn1 in ciliary trafficking and virus uncoating has been clearly established, the binding of Trn1 in these processes and the related molecular mechanisms remain largely unknown. As for the recognition of non-PY-NLSs, detailed biochemical and structural studies would for instance greatly help understand how Trn1 interacts with substrates destined to ciliary trafficking.

Nowadays, Trn1 attracts a lot of attention for its clear protective activity in RNA-binding protein phase separation and maturation of their inclusions in cells. Overexpression of Trn1 reverses aberrant phase separation *in vitro* and in cells (Guo et al., 2018; Hofweber et al., 2018; Yoshizawa et al., 2018). Trn1 restores FUS-mRNA targets expression in ALS-derived fibroblast and rescues impaired protein synthesis in axon terminals (Guo et al., 2018; Qamar et al., 2018). Studies in fly models showed an increase in lifespan, when mutated FUS was co-expressed with Trn1 in motor neurons, and complete rescue of MSP-linked hnRNPA2 mutation (Guo et al., 2018). Together, these recent studies indicate that Trn1 might have a direct therapeutic utility in fatal neurodegenerative diseases.
